# Use of diagnostic likelihood ratio of outcome to evaluate misclassification bias in the planning of database studies

**DOI:** 10.1186/s12911-022-01757-1

**Published:** 2022-01-21

**Authors:** Yoichi Ii, Shintaro Hiro, Yoshiomi Nakazuru

**Affiliations:** Biometrics and Data Management, Development Japan, Pfizer R&D Japan, 3-22-7, Yoyogi, Shibuya-ku, Tokyo, 151-8589 Japan

**Keywords:** Claims, Database study, Healthcare, Likelihood ratio, Outcome, Predictive values, Risk ratio, Sensitivity, Specificity, Validation study

## Abstract

**Background:**

The diagnostic likelihood ratio (DLR) and its utility are well-known in the field of medical diagnostic testing. However, its use has been limited in the context of an outcome validation study. We considered that wider recognition of the utility of DLR would enhance the practices surrounding database studies. This is particularly timely and important since the use of healthcare-related databases for pharmacoepidemiology research has greatly expanded in recent years. In this paper, we aimed to advance the use of DLR, focusing on the planning of a new database study.

**Methods:**

Theoretical frameworks were developed for an outcome validation study and a comparative cohort database study; these two were combined to form the overall relationship. Graphical presentations based on these relationships were used to examine the implications of validation study results on the planning of a database study. Additionally, novel uses of graphical presentations were explored using some examples.

**Results:**

Positive DLR was identified as a pivotal parameter that connects the expected positive-predictive value (PPV) with the disease prevalence in the planned database study, where the positive DLR is equal to sensitivity/(1-specificity). Moreover, positive DLR emerged as a pivotal parameter that links the expected risk ratio with the disease risk of the control group in the planned database study. In one example, graphical presentations based on these relationships provided a transparent and informative summary of multiple validation study results. In another example, the potential use of a graphical presentation was demonstrated in selecting a range of positive DLR values that best represented the relevant validation studies.

**Conclusions:**

Inclusion of the DLR in the results section of a validation study would benefit potential users of the study results. Furthermore, investigators planning a database study can utilize the DLR to their benefit. Wider recognition of the full utility of the DLR in the context of a validation study would contribute meaningfully to the promotion of good practice in planning, conducting, analyzing, and interpreting database studies.

**Supplementary Information:**

The online version contains supplementary material available at 10.1186/s12911-022-01757-1.

## Background

The use of healthcare-related databases (DBs) for pharmacoepidemiology research has expanded in recent years [1]. A PubMed search found a nearly six-fold increase in the number of publications related to DB studies and administrative claims data from the decade spanning 2001‒2010 to 2011‒2020^†^. A rapid increase has also been reported in the Asia‒Pacific region, where such databases have become widely available in recent years [2]. [† Search query for Title/Abstract: ("database study" OR "database studies") AND ("claims" OR "administrative"); Search date: 06 JAN 2021].

In such times of change, it is important to make renewed efforts to promote good practice in the planning, conduction, analysis, and interpretation of DB studies. Advancing the understanding of outcome validation studies is an essential part of these efforts. Outcome validation studies are particularly important for DB studies based on secondary use DBs, such as administrative claim DBs. This paper focuses on how to utilize the existing validation studies to inform and evaluate the design of a new claim-based DB study in its planning phase. One possible conclusion from such evaluation is that there is not enough information to proceed with confidence, leading to a decision to conduct a new validation study. The steps after the conduct of the DB study, which may include bias adjustments using the data from the validation studies, are out of the scope of this paper.

In a claim-based DB study, the source information typically includes diagnosis, drug prescription, and medical procedure records from an administrative claims DB. The outcome of interest is defined by a specific combination of these records. When the source DB is the electronic medical record (EMR), such a combination of records is sometimes referred to as the “EMR-derived phenotype algorithm” [3]. In this paper, we will use the term “phenotype algorithm” or simply “algorithm” when there is no confusion. Even a well-considered algorithm is not perfect in identifying the true occurrence (or lack of occurrence) of an outcome. Thus, an “outcome validation study” is conducted to characterize the degree of imperfection of the algorithm. More specifically, a validation study characterizes the relationship between the proposed algorithm and a “gold standard” evaluation.

In addition to outcomes, the validation target may include exposure variables (e.g., the use of specific drugs), selection variables (e.g., the diagnosis of a specific disease), or confounder variables. To refer to these wider usages, a generic term “validation study” is used. Some general references related to validation studies are available [4–9]. For common outcomes, systematic reviews of validation studies are available [6, 10–17].

The “diagnostic likelihood ratio” (DLR) and its utility are well-known in the field of medical diagnostic testing, such as screening tests for specific diseases [18–21]. However, its use in the context of a validation study seems to be limited. We found only two such examples: Barbhaiya et al. [22] and Shrestha et al. [23]. Both used DLR as a summary measure to characterize the target phenotype algorithms. In this paper, we explored additional usages for the DLR. Specifically, we examined the use of DLR in the assessment of bias during the planning of a comparative cohort DB study. We consider that wider recognition of the full utility of the DLR will enhance the practices surrounding DB studies, including those during the reporting of outcome validation studies and the planning of a new DB study.

## Methods

### Outcome validation study

Typically, a validation study is conducted on a random sample from an entire population of subjects. For clarity, we refer to the random sample as “validation study sample” and to the entire population as the “validation study population.” A hypothetical summary of a validation study result is shown in Table 1 (adapted from Figure. 37.1 of Ritchey et al. [6]). The rows represent the outcomes (“positive” or “negative”) as identified by the proposed phenotype algorithm. The columns represent the phenotype or the true disease status (with or without disease) based on the gold standard. For example, N_A_ represents the number of subjects who are identified as positive by the algorithm among those who truly have the disease. N_B_, N_C_, and N_D_ are defined analogously.

**Table 1:** Summary of a typical validation study result (Adapted from Figure 37.1 of Ritchey et al. [6].)

Outcome based on claims data algorithm (phenotype algorithm)	“True” disease status based on gold standard (phenotype)	
	With disease (D+)	Without disease (D−)
Positive (O+)	N_A_ (true positive)	N_B_ (false positive)
Negative (O−)	N_C_ (false negative)	N_D_ (true negative)

N = total number of subjects in the validation study sample (N = N_A_ + N_B_ + N_C_ + N_D_)

N_A_, N_B_, N_C_, N_D_ = number of subjects in each cell

Sensitivity = N_A_/(N_A_ + N_C_)

Specificity = N_D_/(N_B_ + N_D_)

Disease prevalence = (N_A_ + N_C_)/N

Sensitivity and specificity are two fundamental measures of misclassification. Sensitivity is the proportion of subjects identified by the algorithm as positive among those who truly have the disease, i.e., N_A_/(N_A_ + N_C_). Specificity is the proportion of subjects identified by the algorithm as negative among those who are truly without the disease, i.e., N_D_/(N_B_ + N_D_). The disease prevalence in the validation study sample is (N_A_ + N_C_)/N, where N is the total number of subjects in the sample.

The following equations give the relationship between positive and negative DLR and the two misclassification measures.

$$\begin{gathered} \text{Positive\,diagnostic\,likelihood\,ratio} \left( {\text{DLR}^{ + } } \right) = \text{sensitivity}/\left( {1 - \text{specificity}} \right) \hfill \\ \text{Negative\,diagnostic\,likelihood\,ratio} \left( {\text{DLR}^{ - } } \right) = \left( {1 - \text{sensitivity}} \right)/\text{specificity} \hfill \\ \end{gathered}$$

If an appropriate sampling design is employed, the validation study sample can be used to estimate the sensitivity, specificity, and DLR of the validation study population. The precision of the point estimate of each measure can be quantified by their respective confidence intervals (CI).

We now introduce the notation shown in Table 2. First, let Pr(D+;S) denote the probability that a subject truly has the disease (D+) in a population of interest S. If we consider a randomly sampled subject from S, then the probability that a subject has the disease is simply the proportion of subjects with the disease in S. If S is the validation study population S_VS_, then Pr(D+;S_VS_) is the disease prevalence of the validation study population. Next, let Pr(O+|D+;S) denote the probability that a subject’s outcome is positive (O+) according to the algorithm in a subset of S with the disease. The expression Pr(X|Y;S) denotes the conditional probability of X in a subset of S in which Y is true. Thus, Pr(O+|D+;S_VS_) is the probability of a positive outcome in a subset of the validation study population with the disease, which is simply the sensitivity in the validation study population. Analogously, Pr(O−|D− ;S_VS_) is the specificity in the validation study population.

**Table 2:** Notations for prevalence, sensitivity, and specificity

Notation	Interpretation
Pr(D + ; S)	Probability of true disease (D+) in population S [**prevalence**]
Pr(O +|D + ; S)	Probability of “positive” outcome based on the algorithm (O+) in a subset of S with true disease (D+) [**sensitivity**]
Pr(O −|D − ; S)	Probability of “negative” outcome based on the algorithm (O−) in a subset of S without true disease (D−) [**specificity**]

### Comparative cohort database study

In the following, we envision a DB study planning consisting of 4 main steps. The 1st step is to formulate the research question and consider possible study design and database options for the DB study. We assumed this step had been completed and that a comparative cohort study based on the claims database was chosen. We also assumed the risk ratio (test versus control group) was chosen as the relative measure. The 2nd step is to search for relevant validation studies and extract usable information such as sensitivity, specificity, and other performance measure values. The 3rd step is to consider possible values, or a range of possible values, for the risk of the outcome event in the control group based on historical information (e.g., clinical trials, observational studies). Also, there is likely to be a target risk ratio value for the DB study. Such evaluations are commonly conducted in sample size and power calculations for the DB study. The 4th step is to evaluate the impact of the performance measures on the bias of risk ratio and other features of the planned DB study.

#### Positive-predictive values

In a comparative cohort DB study, we wish to infer the true state of disease based on the proposed claims-based algorithm. Because the algorithm is imperfect, as characterized by the validation study results, we need to understand how it performs when applied to the DB study. Two such measures of performance are the positive-predictive value (PPV) and the negative-predictive value (NPV) [6]. In the developments below, estimates of sensitivity, specificity, and disease prevalence are assumed to be available from past validation studies or other sources. Additionally, as before, we distinguish the terms “DB study sample” and “DB study population.”

PPV is the probability that a subject identified by the algorithm as positive truly has the disease. Using Bayes’ theorem from probability theory [21, 24], the PPV of the algorithm when applied to the DB study population (PPV_DB_) can be expressed as follows, where P_DB_ is the disease prevalence of the DB study population:

1A $$\begin{aligned} \text{PPV}_{\rm DB} & = \Pr \left( {\text{D} + |\text{O} + ;\text{S}_{\rm DB} } \right) \\ & = \frac{{\Pr \left( {\text{O} + |\text{D} + ;\text{S}_{\rm DB} } \right)\Pr \left( {\text{D} + ;\text{S}_{\rm DB} } \right)}}{{\Pr \left( {\text{O} + |\text{D} + ;\text{S}_{\rm DB} } \right)\Pr \left( {\text{D} + ;\text{S}_{\rm DB} } \right) + \Pr \left( {\text{O} + |\text{D} - ;\text{S}_{\rm DB} } \right)\Pr \left( {\text{D} - ;\text{S}_{\rm DB} } \right)}} \cdots \text{Bayes'\, theorem} \\ & = \frac{{\text{Sensitivity} \cdot \,\text{P}_{\rm DB} }}{{\text{Sensitivity} \cdot \,\text{P}_{\rm DB} + \left( {1 - \text{Specificity}} \right)\left( {1 - \text{P}_{\rm DB} } \right)}} \\ \end{aligned}$$

1B $$= \frac{{\text{DLR}^{ + } \cdot \,\text{P}_{\rm DB} }}{{\text{DLR}^{ + } \cdot \,\text{P}_{\rm DB} + \left( {1 - \text{P}_{\rm DB} } \right)}}.$$

Equation 1A follows from the previous line because sensitivity and specificity are assumed not to depend on the population so that Pr(O+|D+;S_DB_) = Pr(O+|D+;S_VS_) and Pr(O−|D− S_DB_) = Pr(O−|D− ;S_VS_). In practice, the plausibility of this assumption should be justified [25]. Equation 1B is obtained by dividing the numerator and denominator by the term (1 − Specificity). In many validation studies, an estimate of PPV for the validation study itself (PPV_VS_) is reported. The population value of PPV_VS_ is obtained by replacing P_DB_ in Eq. 1A with the disease prevalence of the validation population (P_VS_). It is noted that the usual estimate of PPV_VS_ (= N_A_/(N_A_ + N_B_)) can be obtained by substituting the estimates of the DLR^+^ and P_VS_ from the validation study into Eq. 1B.

By solving Eq. 1B for the DLR^+^ and by noting that the equation holds for either the validation study or the DB study population, another useful expression for the DLR^+^ is obtained:

2 $$\text{DLR}^{ + } = \left( {\frac{{\text{PPV}_{\rm VS} }}{{1 - \text{PPV}_{\rm VS} }}} \right)\bigg/\left( {\frac{{\text{P}_{\rm VS} }}{{1 - \text{P}_{\rm VS} }}} \right) = \left( {\frac{{\text{PPV}_{\rm DB} }}{{1 - \text{PPV}_{\rm DB} }}} \right)\bigg/\left( {\frac{{\text{P}_{\rm DB} }}{{1 - \text{P}_{\rm DB} }}} \right) = \frac{\text{Post - test odds}}{{\text{Pre - test odds}}}.$$

In the terminology of diagnostic tests, DLR^+^ is equal to the ratio of “post-test odds” to the “pre-test odds” [18, 19]. Pre-test odds is the odds of disease (D+), and post-test odds is the odds of disease when the test result is positive (in the current case, when the ocome is O+). Under the current assumption, the DLR^+^ is invariant between validation and DB studies.

Analogous developments for the NPV are possible, where the DLR^−^ plays the corresponding role.

#### Relative measures of risk

We now examine the impact of misclassifications on relative measures of risk, namely, the risk ratio (RR). As stated by Ritchey et al., the ultimate criterion for the importance of misclassification is the degree of bias exerted on relative measures of risk [6].

Let N_TES_ and N_CON_ indicate the sample sizes of the test and control (referent) groups of a hypothetical cohort DB study, respectively. Similarly, let X_TES_ and X_CON_ indicate the corresponding number of subjects with the true disease, which are assumed to be known for this hypothetical situation. The expected numbers of positive outcomes based on the algorithm and the corresponding risk expressions are given in Table 3. Table 3 assumes that sensitivity and specificity are invariant between the test and control groups. For applications in actual DB studies, the plausibility of this “non-differential misclassification error” should be justified.

**Table 3:** True and expected number of positive outcomes, risks, and risk ratio

Group	Sample size	Number of true positives	Expected number of positive outcomes identified by the algorithm^a^
Control	N_CON_	X_CON_	E_CON_ = X_CON_Se + (N_CON_ − X_CON_)(1 − Sp)
Test	N_TES_	X_TES_	E_TES_ = X_TES_Se + (N_TES_ − X_TES_)(1 − Sp)

Group		True risk	Expected risk based on the algorithm
Control		R_CON_ = X_CON_/N_CON_	E_CON_/N_CON_ = R_CON_Se + (1 − R_CON_)(1 − Sp)
Test		R_TES_ = X_TES_/N_TES_	E_TES_/N_TES_ = R_TES_Se + (1 − R_TES_)(1 − Sp)

Relative measure		True relative measure	Expected relative measure
Risk ratio (RR)		RR_TRUE_ = R_TES_/R_CON_	RR_EXP_ = (E_TES_/N_TES_)/(E_CON_/N_CON_)

Numbers of positive outcomes are those expected under the non-differential misclassification assumption. The numbers include both true- and false-positives based on the algorithm.

^a^Se = sensitivity, Sp = specificity

Using the risk expressions in Table 3, we can write the expected RR in terms of the true RR, as shown in Eq. 3, where RR_EXP_ is the expected RR, RR_TRUE_ is the true RR, and R_CON_ is the true disease risk of the control group in the DB study:

3 $$RR_{EXP} = RR_{TRUE} + \frac{{1 - RR_{TRUE} }}{{R_{CON} \cdot \left( {DLR^{ + } - 1} \right) + 1}}.$$

The details of the derivation are shown in Appendix A (Additional file 1). The term $\left( {1 - RR_{TRUE} } \right)/\left\{ {R_{CON} \cdot \left( {DLR^{ + } - 1} \right) + 1} \right\}$ is the bias of the RR_EXP_ relative to the RR_TRUE_. If the RR_TRUE_ is greater than 1, then the bias term is always negative in this “ideal” situation (see Appendix B, Additional file 1). In real-life situations, there may be other sources of bias so that the overall bias may not be negative [6, 26].

All calculations were performed and graphs were generated using R version 3.6.1 [27].

## Results

### Positive-predictive values

**Fig. 1:**
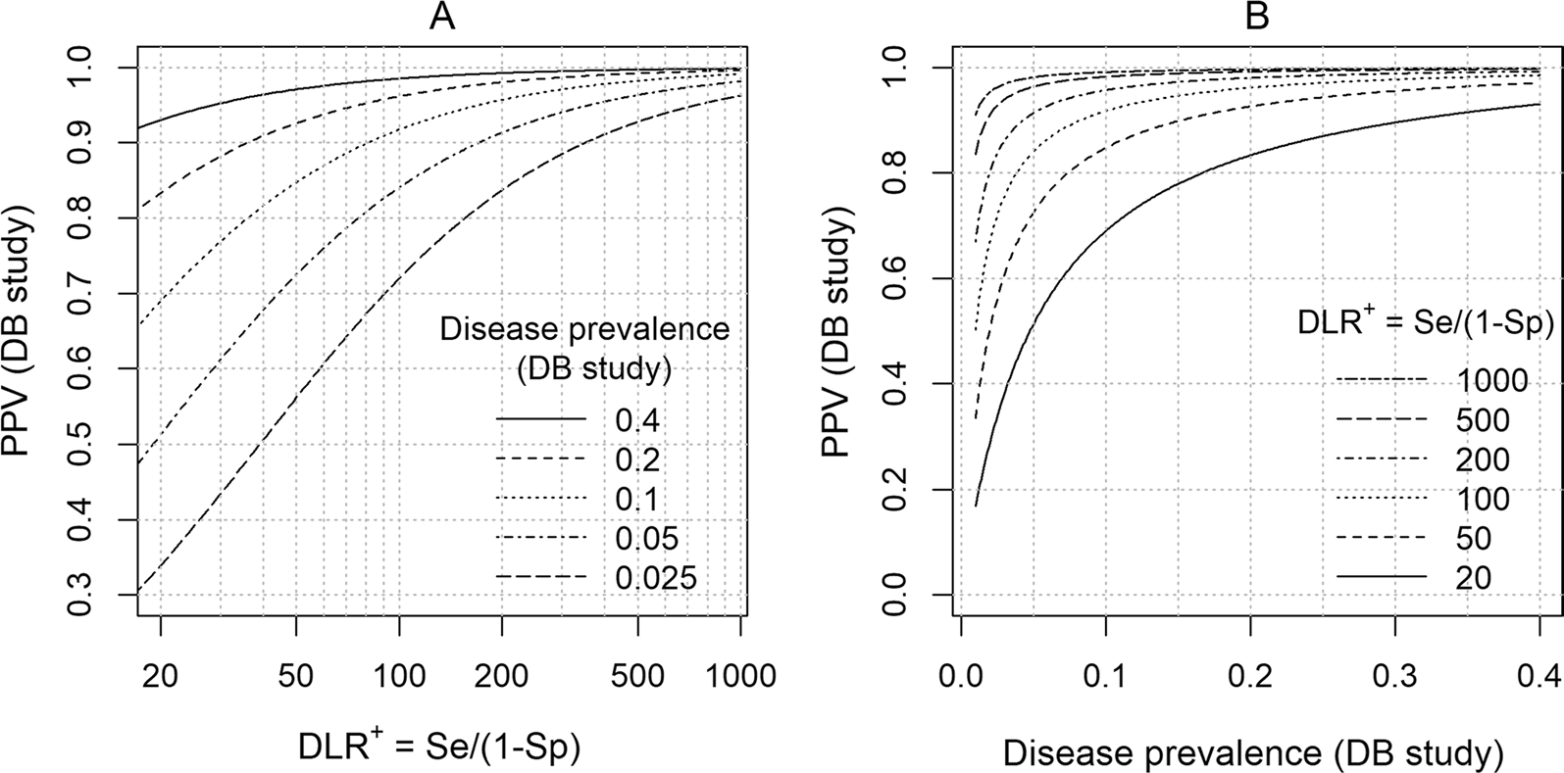
Expected PPV of the DB study as a function of DLR^+^ and disease prevalence. **A** Positive-predictive value (PPV) of the database (DB) study is plotted against positive likelihood ratio (DLR^+^). Each line represents a fixed value of disease prevalence of the DB study population. A hypothetical range of disease prevalence values is shown (0.025–0.4). **B** Expected PPV is plotted against a hypothetical range of the disease prevalence of the DB study. Each line represents a fixed value of DLR^+^. A range of values for the DLR^+^ is shown (20–1000). Both plots are based on Eq. 1B

Figure 1A displays the expected PPV of the DB study as a function of a DLR^+^ and the disease prevalence of the DB study population. A hypothetical range (0.025‒0.4) is graphed for the disease prevalence in the DB study population. For each value of the disease prevalence, the expected PPV of the DB study increases with increasing values of DLR^+^. Figure 1B gives an alternative display format in which the x-axis is the disease prevalence, and each line represents a value of the DLR^+^. For each DLR^+^ value, the expected PPV of the DB study increases with increasing disease prevalence. If the disease prevalence of the DB study population is equal to that of the validation study, then the PPVs are also expected to be equal. It follows that if the disease prevalence of the DB study is likely to be lower than that in the validation study, then the expected PPV of the DB study would be lower than that in the validation study.

**Fig. 2:**
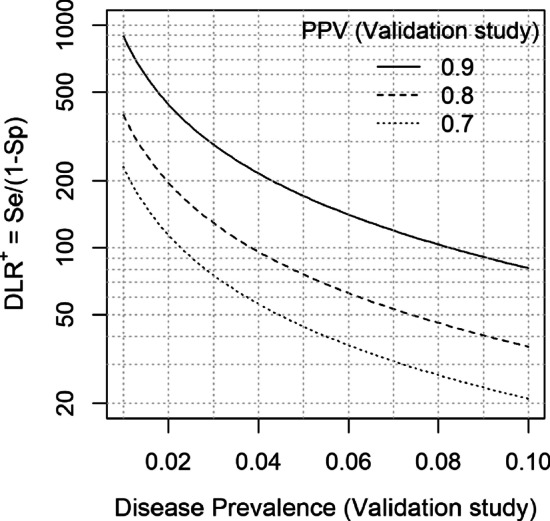
DLR^+^ as a function of disease prevalence of the validation study population. Positive diagnostic likelihood ratio (DLR^+^) is plotted against disease prevalence of the validation study population. Lines are drawn for selected values of positive-predictive value (PPV) for the validation study. The plot is based on Eq. 2 for the validation study population

In many validation studies, sensitivity and specificity are not available, and only PPVs are reported. Thus, previously mentioned assessment methods are not applicable. However, a plausible range of DLR^+^ can be ascertained by using Eq. 2. Figure 2 shows DLR^+^ as a function of disease prevalence of the validation study (P_VS_) for selected values of the PPV for the validation study (PPV_VS_). Suppose a plausible range of P_VS_ is 0.04‒0.06, based on information from the validation study or other sources, and the PPV_VS_ is 0.8 according to the validation study. From Fig. 2, the corresponding range of DLR^+^ is approximately 63‒96. If desired, a range of values for PPV_VS_ may be considered to account for the precision of the estimate. Once the value of DLR^+^ is in hand, one can refer to Fig. 1, as before.

### Relative measures of risk

**Fig. 3:**
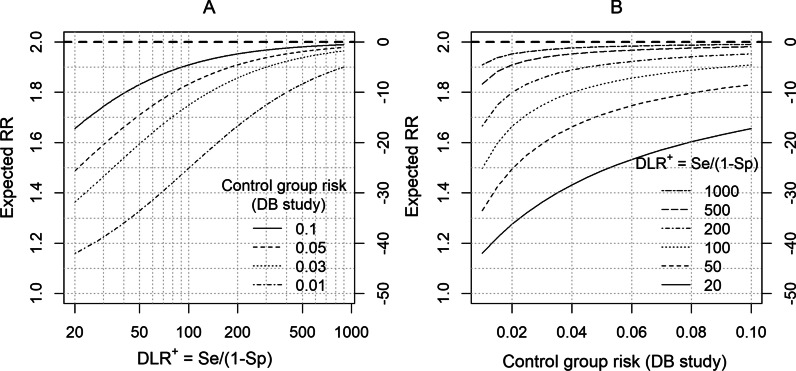
Expected RR as a function of DLR^+^ and the true disease risk. Expected risk ratio (RR) of the database (DB) study is shown as a function of positive diagnosis likelihood ratio (DLR^+^) and the true disease risk of the control (referent) group of the DB study. The true RR is set to 2.0. **A** Expected RR is plotted against DLR^+^. A hypothetical range of values for the true disease risk of the control group is shown (0.01–0.1). **B** Expected RR is plotted against the true disease risk of the control group. A range of values for the DLR^+^ is shown (20–1000). The right axis of each plot displays the scales in terms of % bias relative to the true RR. Both plots are based on Eq. 3. Figure X1 (Additional file 1) gives a more traditional display in which sensitivity and specificity are considered separately

Figure 3A displays the RR_EXP_ as a function of the DLR^+^ and the true disease risk of the control group of the DB study. For illustrative purposes, the RR_TRUE_ is set to 2.0, and a hypothetical range of values (0.01‒0.1) for the true disease risk of the control group (R_CON_) is graphed. For each value of the control group’s risk, the degree of bias decreases with increasing values of the DLR^+^. Figure 3B gives an alternative display format in which the x-axis is the control group risk. For each value of the DLR^+^, the degree of bias decreases with increasing values of the control group risk. Figure 3A and B permit a more compact and transparent way of visualizing the relationship between the expected RR and the control group risk of the DB study, as compared with a traditional display format shown in Appendix Figure X1 (Additional file 1).

### Use examples

Published examples of the DLR in the context of outcome validation studies are rare. Barbhaiya et al. (2017) conducted a validation study of claim-based phenotype algorithms for identifying the diagnosis of avascular necrosis [22]. In their paper, the DLR^+^ was used as a summary measure, along with the sensitivity, specificity, and PPV. Shrestha et al. (2016) conducted a systematic review of administrative data-based phenotype algorithms for the diagnosis of osteoarthritis [23]. In their review, the DLR^+^ was included as a summary measure of the phenotype algorithms, along with sensitivity, specificity, and expected PPV values at three hypothetical values of the disease prevalence. We recommend a routine inclusion of DLR in a validation study report whenever it is computable.

As a further illustration of the use of the DLR^+^, we provide two artificial examples based on data from a systematic review by McCormick et al. [17]. The review identified 30 studies on administrative data-based phenotype algorithms for the diagnosis of acute myocardial infarction (MI). We envision planning a DB study with acute MI outcomes.

In the first artificial example, we selected three studies that reported sensitivity, specificity, PPV, and NPV: Kennedy et al. [28], Pladevall et al. [29], and Austin et al. [30]. Many studies in the review reported only PPVs. Table 4 provides a summary of the three studies. We supplemented the DLR^+^ and its 95% confidence interval (CI), which were not included in either the systematic review or the original reports. In addition, we calculated two features of the planned DB study that would be expected under specific assumptions. The first feature is the expected PPV when the prevalence of acute MI is assumed to be 0.05 in the planned DB study. The second feature is the relative bias of the RR when the control group’s risk of acute MI and the true RR are assumed to be 0.03 and 2.0, respectively. The relative bias is defined as the bias divided by the true RR multiplied by 100%. The assumptions were chosen for illustrative purposes.

**Table 4:** Use example of DLR^+^ in validation studies and in planning of a DB study

Cell counts	Kennedy [28]		Pladevall [29]		Austin [30]	
N	20,386		5329		58,816	
N_A_	67		401		20,048	
N_B_	43		333		2594	
N_C_	4		95		2521	
N_D_	20,272		4500		33,653	

Validation study	Estimate	95%CI^d^	Estimate	95%CI	Estimate	95%CI
Sensitivity	0.944	0.862, 0.984	0.808	0.771, 0.842	0.888	0.884, 0.892
Specificity	0.998	0.997, 0.998	0.931	0.924, 0.938	0.928	0.926, 0.931
Prevalence	0.003	0.003, 0.004	0.093	0.085, 0.101	0.384	0.380, 0.388
PPV^a^	0.609	0.511, 0.701	0.546	0.509, 0.583	0.885	0.881, 0.890
NPV^a^	1.000	0.999, 1.000	0.979	0.975, 0.983	0.930	0.928, 0.933
DLR^+a^	445.8	329.0, 604.2	11.7	10.5, 13.1	12.4	12.0, 12.9

DB study	Expected	Range^e^	Expected	Range	Expected	Range
PPV at 0.05^b^	0.959	0.945, 0.970	0.382	0.356, 0.409	0.395	0.386, 0.404
Relative bias of RR(%) at 0.03^c^	− 3.49	− 4.61, − 2.62	− 37.8	− 38.9, − 36.7	− 37.2	− 37.6, − 36.9

Three validation studies included in a systematic review by McCormick et al. are utilized [17].

^a^Positive predictive value (PPV), negative predictive value (NPV), positive likelihood ratio (DLR^+^)

^b^Expected PPV of the planned database (DB) study at population prevalence of 0.05. The calculation was based on Eq. 1B.

^c^Expected relative bias of risk ratio (RR) at DB study control group risk of 0.03. The relative bias is defined as bias/true RR × 100%, where the true RR is assumed to be 2. The calculation was based on Eq. 3.

^d^95% confidence interval (CI): Exact method of Clopper-Pearson [31] was use for sensitivity, specificity, PPV, and NPV. Log-transformed approximate method of Katz was used for DLR^+^ [32]. R packages “binom” [33] and “DescTools” [34] were used in the calculation.

^e^Range corresponding to the 95% CI of DLR^+^

**Fig. 4:**
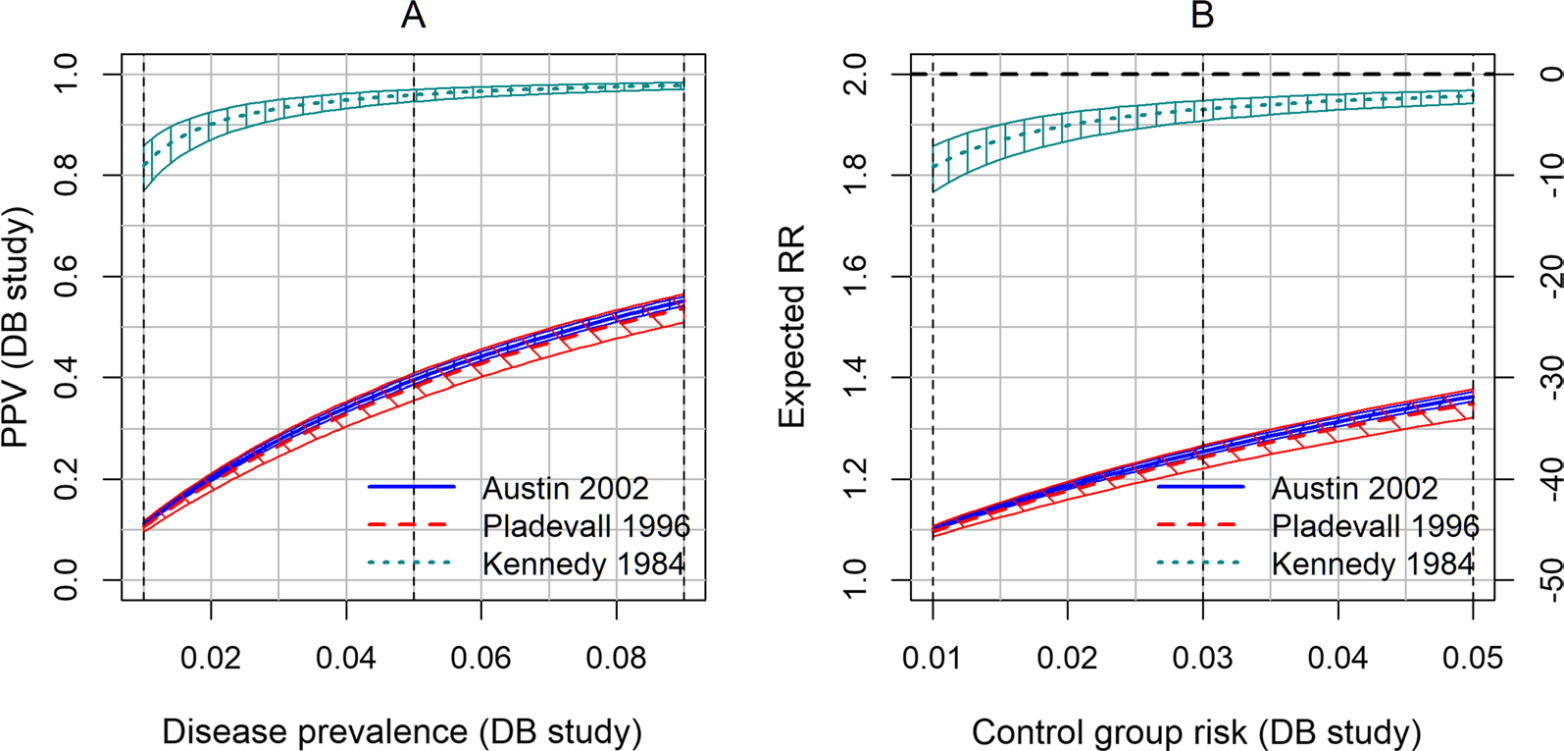
An example of application of Eqs. 1B and 3 to data from actual validation studies. Validation studies by Austin [30], Pladevall [29] and Kennedy [28] were selected from the systematic review by McCormick et al. [17]. **A** Expected positive-predictive value (PPV) of the planned database (DB) study is plotted against the disease prevalence of the DB study. **B** Expected risk ratio (RR) of the planned DB study is plotted against the control group risk of the DB study. The right axis is in terms of relative bias scale. In each panel, center, lower and upper lines for each study correspond to the point estimate and lower and upper bounds of 95% confidence interval of DLR^+^

The DLR^+^ value for the Kennedy study is nearly 40 times greater than that of the other two studies (Table 4). This translates to a large difference in the expected PPV and bias of the RR between Kennedy and the other studies. Figure 4A displays the expected PPV in the DB study for a hypothetical range of disease prevalence, which is set to 0.01‒0.09 for our illustration. For the Kennedy study, the expected PPV at a disease prevalence of 0.05 is 0.959, which contrasts with values below 0.4 for the other two studies (Table 4 and Fig. 4A). Figure 4B displays the expected bias of the RR for a plausible range of the control group’s risk, which is assumed to be 0.01‒0.05 for our illustration (true RR is set to 2.0). For the Kennedy study, the bias of RR is − 3.49% at a control group risk of 0.03, which contrasts with values less than − 37% for the other two studies (Table 4 and Fig. 4B). Additionally, the disease prevalence is 0.003 for the Kennedy study, which is notably lower than that of the other two studies (Table 4). Thus, planning for the DB study is greatly affected by the choice of validation studies. In actual applications, one needs to evaluate various features of the validation studies carefully and select those studies that are most relevant for the planned DB study. The validation study features to be scrutinized might include the study population, the “gold standard” criteria, and the outcome definition. Also, in actual applications, the range of parameters such as the disease prevalence and control group risk should be judiciously selected by each investigator based on past information and to cover relevant expected scenarios in the planned DB study.

**Fig. 5:**
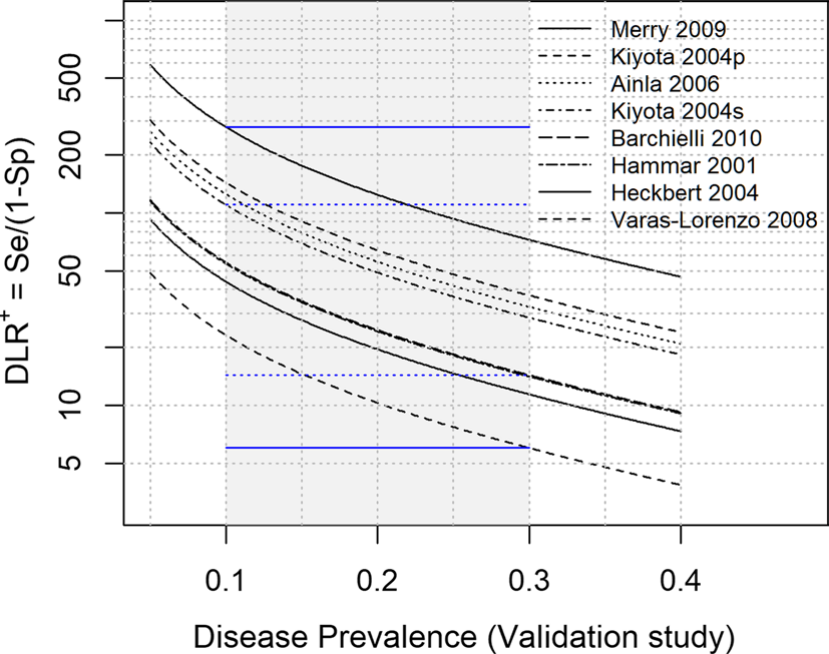
An example of application of Eq. 2 to algorithms from validation studies. Positive likelihood ratio (DLR^+^) is plotted against the disease prevalence of the validation study. Each line is drawn corresponding to the reported PPV value for each algorithm. Seven studies (eight algorithms) were selected from Fig. 2A of the systematic review by McCormick et al. [17]. Ordering of the algorithms in the legend corresponds to the order of lines in the graph. Included algorithms and the reported PPVs are: Merry 2009 (0.9688), Kiyota 2004p (primary diagnosis) (0.9411), Ainla 2006 (0.933), Kiyota 2004s (primary or secondary diagnosis) (0.9245), Barchielli 2010 (0.8602), Hammar 2001 (0.8583), Heckbert 2004 (0.8302), and Varas-Lorenzo 2008 (0.7202). The gray band indicates disease prevalence between 0.1 and 0.3. Horizontal blue solid lines indicate DLR^+^ values that are consistent with all eight algorithms; blue dotted lines indicate DLR^+^ values that are consistent with the “median” algorithm

The second example involves a case in which only PPVs are reported. In this case, the previous type of assessment is not applicable. McCormick et al. [17] reported a systematic difference in PPV values between studies with and without cardiac troponin measurement as a part of the “gold standard.” For this illustration, we considered eight phenotype algorithms from seven studies in Fig. 2A of McCormick et al. [17], whose gold standard criteria included cardiac troponin measurements. Figure 5 plots the DLR^+^ against the disease prevalence for the reported PPV value for each algorithm. Each line is drawn based on the relationship in Eq. 2. A wide range of disease prevalence is displayed to consider various possibilities.

A detailed examination of each validation study and the related sources may provide a hint on a narrower plausible range for the disease prevalence. Suppose that this plausible range is taken to be 0.1‒0.3 (shown by the shaded region in Fig. 5). Next, consider a freely moving horizontal line moving up from the bottom of the figures. The horizontal line crosses the first algorithm (Varas-Lorenzo, 2008) at the disease prevalence of 0.3 (DLR^+^  = 6). As the horizontal line continues to move up, it will cross multiple algorithms. Analogously, a horizontal line moving down from the top of the figure crosses the first algorithm (Merry, 2009) at the disease prevalence of 0.1 (DLR^+^  = 279). Thus, the range of the DLR^+^ values that is consistent with all eight algorithms is 6‒279; this range is indicated by a pair of horizontal blue solid lines in Fig. 5. In actual applications, this range for DLR^+^ may be too wide, and algorithm selection may need to be refined further. One idea to narrow the range might be to consider DLR^+^ values that are consistent with the “median” algorithm, which, in this case, are the two central algorithms (i.e., Kiyota 2004s and Barchielli 2010). A pair of horizontal blue dotted lines in Fig. 5 indicates such a range (Note: the Barchielli 2010 and Hammar 2001 algorithms nearly overlap in Fig. 5). Once a plausible range of DLR^+^ value is determined based on assessments such as above, one can compute the corresponding range for the expected RR using Eq. 3.

## Discussion

In this paper, we investigated the utility of the DLR in the context of an outcome validation study. Positive DLR was identified as a pivotal parameter that connects the expected PPV with the disease prevalence in the planned DB study, where the positive DLR is equal to sensitivity/(1-specificity). Moreover, positive DLR emerged as a pivotal parameter that links the expected RR with the disease risk of the control group in the planned DB study.

The importance of thorough sensitivity analyses after the completion of a DB study is well established [6, 35–38]. In contrast, there has been less focus on what can be done to improve the planning of a DB study. During the planning phase, careful assessments of outcome definitions and other elements of the study design should be conducted. Toward this end, the DLR provides a transparent and informative summary of the relationship between PPVs that can be expected in the planned DB study based on the results of a validation study (Fig. 1). Additionally, the expected degree of bias of the RRs can be characterized clearly (Fig. 3).

There are some limitations to the method described above. As mentioned in “Methods” section, there are assumptions in the derivation of the equations, such as the non-differential misclassification error. The invariance of sensitivity and specificity between the validation study and the DB study populations is another assumption. If assessments of sensitivity to deviations from these assumptions are desired, an investigator can start with an expression such as that in Table 3 and use computer calculations to evaluate performance under any arbitrary settings. In particular, the assumption of non-differential misclassification error requires careful considerations. In addition, extensions to other relative measures such as the risk difference and odds ratio as well as non-binary variables (e.g., continuous, categorical) may be of interest. Finally, although we focused on claim-based DB studies, some features are also relevant for DB studies based on electronic health records.

## Conclusions

Wider recognition of the full utility of the DLR in the context of validation studies will make a meaningful contribution to the promotion of good practice in the planning, execution, analysis, and interpretation of DB studies.

## Supplementary Information

**Additional file 1**. Appendix A, B and Figure X1.

## Data Availability

Data sharing is not applicable to this article as no datasets were generated or analyzed during the current study.

## Abbreviations

CI: Confidence interval
DB: Database
DLR: Diagnostic likelihood ratio
EMR: Electronic medical record
MI: Myocardial infarction
NPV: Negative-predictive value
PPV: Positive-predictive value
RR: Risk ratio
