# Supplementary material for: Use of diagnostic likelihood ratio of outcome to evaluate misclassification bias in the planning of database studies
Source: BMC Med Inform Decis Mak. 2022 Jan 21;22:19. doi: 10.1186/s12911-022-01757-1 (PMC8783524; doi:10.1186/s12911-022-01757-1)
Supplement: Supplementary file 1 — Additional file 1. Appendix A, B and Figure X1. [file 12911_2022_1757_MOESM1_ESM.docx]

**Appendix**

Throughout the appendix, the following notations are used:

- Se: Sensitivity
- Sp: Specificity
- $\mathrm{DLR}^{+}:$ Positive diagnostic likelihood ratio

1. **Derivation of Equation 3**

Let R_TES_ and R_CON_ be the true risk of test and control (referent) groups in the DB study, respectively. Using the expression for the expected risk ratio (RR_EXP_) in Table 3, the following relationship with the true risk ratio (RR_TRUE_) is derived.

$$\begin{matrix} RR_{\mathrm{EXP}} & = & \frac{R_{\mathrm{TES}}\cdot Se+\left( 1-R_{\mathrm{TES}} \right)\left( 1-Sp \right)}{R_{\mathrm{CON}}\cdot Se+\left( 1-R_{\mathrm{CON}} \right)\left( 1-Sp \right)} \\ & = & \frac{R_{\mathrm{TES}}\cdot\left( Se-\left( 1-Sp \right) \right)+\left( 1-Sp \right)}{R_{\mathrm{CON}}\cdot\left( Se-\left( 1-Sp \right) \right)+\left( 1-Sp \right)} \\ & = & \frac{R_{\mathrm{TES}}\cdot\left( \mathrm{DLR}^{+}-1 \right)+1}{R_{\mathrm{CON}}\cdot\left( \mathrm{DLR}^{+}-1 \right)+1} \\ & = & \frac{R_{\mathrm{TES}}}{R_{\mathrm{CON}}}+\left\{ \frac{R_{\mathrm{TES}}\cdot\left( \mathrm{DLR}^{+}-1 \right)+1}{R_{\mathrm{CON}}\cdot\left( \mathrm{DLR}^{+}-1 \right)+1}-\frac{R_{\mathrm{TES}}}{R_{\mathrm{CON}}} \right\} \\ & = & RR_{\mathrm{TRUE}}+\left\{ \frac{\left( \frac{R_{\mathrm{TES}}}{R_{\mathrm{CON}}}\cdot R_{\mathrm{CON}}\cdot\left( \mathrm{DLR}^{+}-1 \right)+1 \right)}{R_{\mathrm{CON}}\cdot\left( \mathrm{DLR}^{+}-1 \right)+1}-\frac{R_{\mathrm{TES}}}{R_{\mathrm{CON}}} \right\} \\ & = & RR_{\mathrm{TRUE}}+\frac{\frac{R_{\mathrm{TES}}}{R_{\mathrm{CON}}}{\cdot R}_{\mathrm{CON}}\cdot\left( \mathrm{DLR}^{+}-1 \right)+1-\frac{R_{\mathrm{TES}}}{R_{\mathrm{CON}}}\left\{ R_{\mathrm{CON}}\cdot\left( \mathrm{DLR}^{+}-1 \right)+1 \right\}}{R_{\mathrm{CON}}\cdot\left( \mathrm{DLR}^{+}-1 \right)+1} \\ & = & RR_{\mathrm{TRUE}}+\frac{1-RR_{\mathrm{TRUE}}}{R_{\mathrm{CON}}\cdot\left( \mathrm{DLR}^{+}-1 \right)+1} \end{matrix}$$

1. **The sign of the bias term in Equation 3**

Consider the denominator term in Equation 3: $R_{\mathrm{CON}}\cdot\left( \mathrm{DLR}^{+}-1 \right)+1$. If this term is positive, then the bias is always negative (positive) when the true risk ratio is >1 (<1). The bias is zero when the true risk ratio is equal to 1. Because both sensitivity and specificity take on values between 0 and 1, $\mathrm{DLR}^{+}\geq0$. Thus, the minimum value of $R_{\mathrm{CON}}\cdot\left( \mathrm{DLR}^{+}-1 \right)+1$ is greater than or equal to $1-R_{\mathrm{CON}}$. It follows that the denominator term is positive if R_CON_<1. The case R_CON_=1 is not of a practical concern.

It is noted that $\mathrm{DLR}^{+}>1$ for any “reasonable” algorithm. This is because $\mathrm{DLR}^{+}=\Pr\left( O+ | D+ \right)/Pr(O+|D-)$ and any “reasonable” algorithm would have a higher probability of positive outcomes when applied to a diseased population compared with a non-diseased population.

**Figure X1. Expected RR of the DB study as a function of sensitivity and specificity**


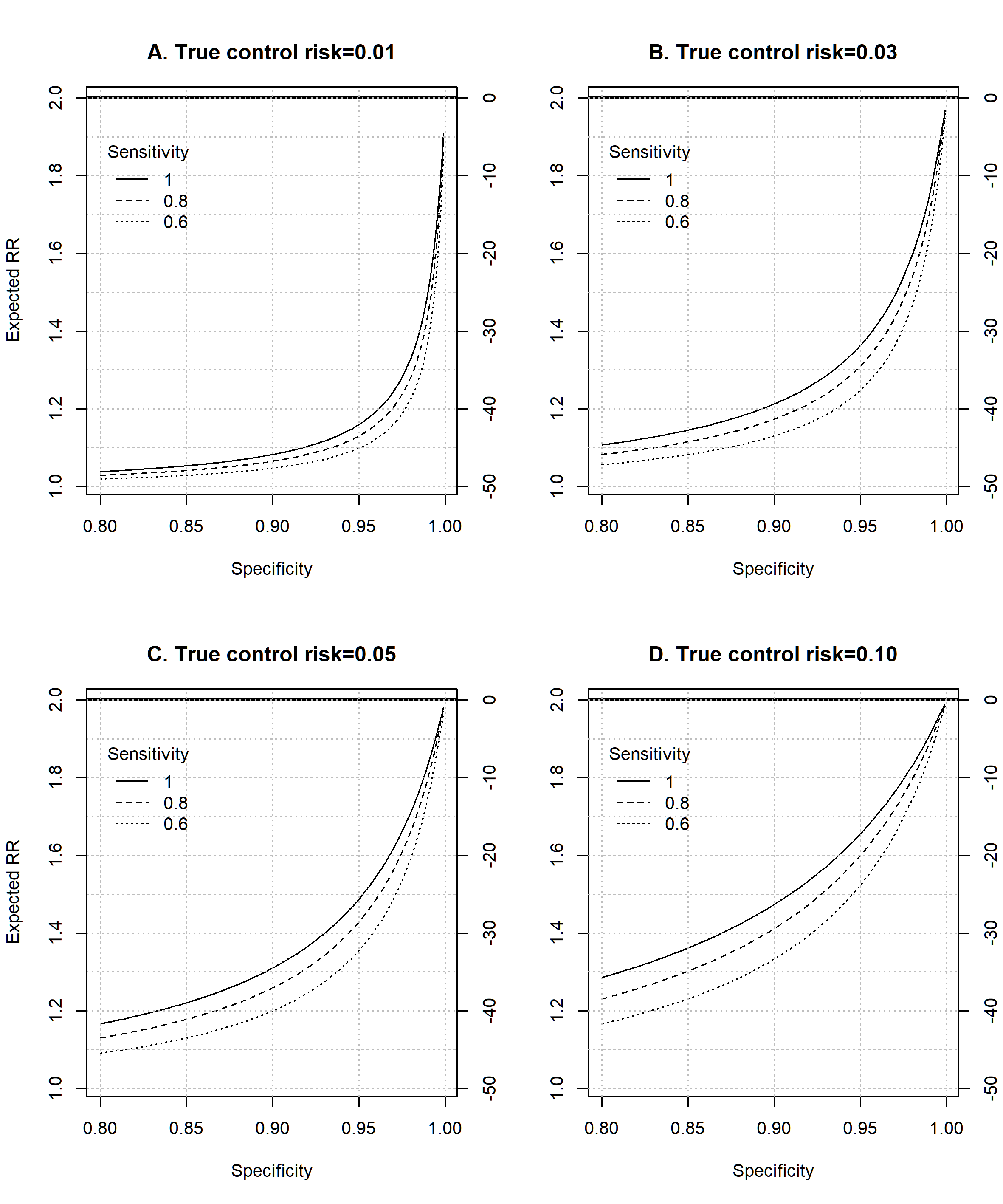


Expected risk ratio (RR) of the database (DB) study is shown as a function of sensitivity and specificity. The true RR is set to 2.0. The true disease risk of the control (referent) group of DB study is: A) 0.01, B) 0.03, C) 0.05, or D) 0.1. The right axis in each plot displays scales in terms of % bias relative to the true RR. Plots are based on Equation 3 and the definition of the positive diagnostic ratio. Figures of this format are given in Figures 1 and 2 of Copeland et al. [1].

[1] K. T. Copeland, H. Checkoway, A. J. McMichael and R. H. Holbrook, "Bias due to misclassification in the estimation of relative risk," *Americal Journal of Epidemiology,* vol. 105, no. 5, pp. 488-495, 1977.
